# Archaeal Nucleic Acid Ligases and Their Potential in Biotechnology

**DOI:** 10.1155/2015/170571

**Published:** 2015-10-01

**Authors:** Cecilia R. Chambers, Wayne M. Patrick

**Affiliations:** Department of Biochemistry, University of Otago, P.O. Box 56, Dunedin 9054, New Zealand

## Abstract

With their ability to catalyse the formation of phosphodiester linkages, DNA ligases and RNA ligases are essential tools for many protocols in molecular biology and biotechnology. Currently, the nucleic acid ligases from bacteriophage T4 are used extensively in these protocols. In this review, we argue that the nucleic acid ligases from Archaea represent a largely untapped pool of enzymes with diverse and potentially favourable properties for new and emerging biotechnological applications. We summarise the current state of knowledge on archaeal DNA and RNA ligases, which makes apparent the relative scarcity of information on *in vitro* activities that are of most relevance to biotechnologists (such as the ability to join blunt- or cohesive-ended, double-stranded DNA fragments). We highlight the existing biotechnological applications of archaeal DNA ligases and RNA ligases. Finally, we draw attention to recent experiments in which protein engineering was used to modify the activities of the DNA ligase from *Pyrococcus furiosus* and the RNA ligase from *Methanothermobacter thermautotrophicus*, thus demonstrating the potential for further work in this area.

## 1. Introduction

DNA and RNA ligases are ubiquitous enzymes that catalyse the formation of phosphodiester bonds between opposing 5′-phosphate and 3′-hydroxyl termini in nucleic acids [1–3]. Their activities are essential for central biological processes, including DNA replication and recombination, rearrangement of immunoglobulin genes, and RNA editing and repair. Their activities* in vitro* have also been exploited in numerous molecular biology protocols, making them critical tools for modern biotechnology.

Together with the RNA capping enzymes and tRNA ligases, DNA and RNA ligases constitute the nucleotidyl transferase superfamily [4]. All of the enzymes in this superfamily catalyse phosphodiester bond formation in a conserved, three-step mechanism that utilises either ATP, GTP, or NAD^+^ as a high-energy cofactor [4–6]. In the first step, nucleophilic attack on the *α*-phosphate of the cofactor by an active site lysine yields a ligase-AMP intermediate. Secondly, the AMP is transferred to the 5′-phosphate of one polynucleotide strand, resulting in an adenylated nucleic acid intermediate (with the active site lysine as the leaving group). Finally, the 3′-hydroxyl group of the second polynucleotide strand attacks the 5′-phosphate of the opposing strand, joining the two strands with a new phosphodiester bond and liberating AMP.

Species from the domain Archaea not only survive, but thrive, under conditions of extreme temperature, salinity, pH, and pressure. Evolution in these extreme environments has resulted in archaeal proteins that have properties of value to biotechnologists, including stability and activity under a range of comparatively harsh* in vitro* conditions. A familiar example is the widespread use of the* Pyrococcus furiosus* DNA polymerase in the Polymerase Chain Reaction (PCR) [7], in which its thermostability and processivity also make it valuable for related protocols such as QuikChange mutagenesis [8, 9]. In this review, we turn the attention to archaeal nucleic acid ligases. We summarise the current state of knowledge about these enzymes, including their existing applications in biotechnology, and we argue that they offer a largely untapped pool of activities for use in “next generation” molecular biology protocols.

## 2. DNA Ligases

*In vivo*, DNA ligases catalyse the formation of phosphodiester bonds at single-stranded nicks in double-stranded DNA. This activity is critical for maintaining genomic integrity during DNA replication, DNA recombination, and DNA excision repair [2, 6]. They are essential in all organisms and they are conventionally classified into two families according to their cofactor specificity. ATP-dependent ligases (EC 6.5.1.1) are typically found in Eukarya, Archaea, and viruses (including bacteriophages), while the NAD^+^-dependent DNA ligases (EC 6.5.1.2) are typically found in bacteria and some eukaryotic viruses. There are, however, exceptions to this rule. Most notably, the archaeal species* Haloferax volcanii* possesses two active DNA ligases: one ATP-dependent (LigA) and the other NAD^+^-dependent (LigN) [10].

DNA ligases are essential for numerous applications in molecular biology and biotechnology. For decades, DNA ligases have been used to construct recombinant DNA molecules (i.e., cloning) and for genetic disease detection using the ligation chain reaction [11]. More recently, the DNA ligase from the bacterium,* Thermus aquaticus* (*Taq*), has become important for Gibson assembly. This is an isothermal, one-pot method for assembling overlapping DNA molecules without the use of restriction enzymes [12]. A number of next generation sequencing methods also depend on DNA ligases [13, 14], either for adapter ligation during sample preparation (e.g., Illumina and 454 sequencing) or for the sequencing reaction itself (SOLiD sequencing).

With its ability to ligate both cohesive- and blunt-ended, double-stranded DNA molecules [15], the most commonly used DNA ligase in biotechnology is the ATP-dependent enzyme from bacteriophage T4. However, it is only weakly active for the ligation of blunt-ended fragments [16] and it is irreversibly inactivated at 65°C. It is also inactive at NaCl concentrations above ~150 mM. We posit that thermostable archaeal DNA ligases could be well suited for use in some or all of the above applications. For example, the ligation chain reaction requires a ligase that is stable at temperatures above 90°C, and Archaea may provide biotechnologists with superior alternatives to* Taq* ligase for Gibson assembly.

## 3. Archaeal DNA Ligases

To date, fewer than 25 archaeal DNA ligases have been characterised to any extent at all, and in general data on them is limited compared with DNA ligases from other domains of life [17, 18].  Table 1 summarises the current state of knowledge and highlights the diverse range of properties possessed by archaeal DNA ligases. Data for T4 DNA ligase are also included, for comparison.

As noted above, DNA ligases are usually classified based on their strict cofactor specificity for either ATP or NAD^+^. Interestingly, a number of archaeal DNA ligases can utilise multiple cofactors. Sequence homology suggested that the DNA ligases from* Thermococcus kodakaraensis, T. fumicolans,* and* T. onnurineus* belong to the ATP-dependent family (EC 6.5.1.1); however,* in vitro* characterisation of each has shown that they are able to use either ATP or NAD^+^ as their cofactor [19–21]. The ATP-dependent DNA ligase from* Sulfophobococcus zilligii* also shows relative activity of 63% when the cofactor is switched from ATP to GTP [22]. Other than the* S. zilligii* DNA ligase, activity with GTP has only been described for RNA capping enzymes [4]. A number of archaeal DNA ligases, including the* S. zilligii* enzyme, are also able to use ADP (Table 1).

One hypothesis for the undifferentiated nucleotide specificities of archaeal DNA ligases is that they have retained a trait from the ancient common ancestor of the ATP- and NAD^+^-dependent enzymes. This ancestor may have used ADP as a cofactor [23], as the ADP moiety is common to both ATP and NAD^+^. However, it has also been noted that direct evidence of ADP utilisation by DNA ligases is minimal [24]. Another proposal is that ATP is comparatively unstable at high temperatures, and this provided the selection pressure for evolution of thermophilic ligases with specificity for alternative cofactors such as ADP and GTP [22].

DNA ligases employ multidomain architectures in order to catalyse phosphodiester bond formation; however, there is variation in the number and identity of the domains they possess [14]. To date, the structures of six archaeal DNA ligases have been solved, from* Archaeoglobus fulgidus* [25],* Pyrococcus furiosus* [26, 27],* Sulfolobus solfataricus* [28],* S. zilligii* [29],* Thermococcus sibiricus* [30], and* Thermococcus* sp. 1519 [31]. Each enzyme comprises three domains: the adenylation domain (AdD), the oligonucleotide-binding domain (OBD), and the N-terminal DNA-binding domain (DBD). The AdD contains the six motifs (I, III, IIIb, IV, V, and VI) that are characteristic of the nucleotidyl transferase superfamily [32]. The AdD and OBD are minimally required for activity and together they are referred to as the catalytic core. The N-terminal DBD is unique to the eukaryotic and archaeal DNA ligases and is thought to play roles in maintaining an active conformation of the catalytic core, as well as distorting the DNA substrate [33].

**Figure 1:**
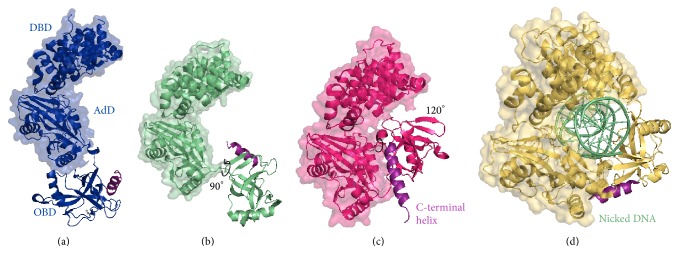
Structures of the DNA ligases from: (a)* Sulfolobus solfataricus* (PDB ID 2HIX); (b)* Thermococcus* sp. 1519 (PDB ID 3RR5); (c)* Pyrococcus furiosus* (PDB ID 2CFM); and (d)* Homo sapiens* (PDB ID 1X9N). The positions of the DNA binding domain (DBD), adenylation domain (AdD), and oligonucleotide binding domain (OBD) are indicated in the* S. solfataricus* structure. While the DBD and AdD occupy equivalent positions in all of the structures, the* S. solfataricus* enzyme shows an open extended conformation of the OBD, the* Thermococcus* sp. 1519 enzyme shows a 90° anticlockwise rotation of the OBD relative to the* S. solfataricus* enzyme (indicated by a black arrow) resulting in the intermediate conformation, and the* P. furiosus* ligase has a 120° rotation exhibiting the closed conformation. Human DNA ligase 1 is shown bound to a nicked DNA substrate (light green). The C-terminal helix of each enzyme is highlighted in purple.

Elucidation of the unbound [34] and DNA-bound [35] structures of the ATP-dependent ligase from* Chlorella* virus has highlighted the importance of large conformational changes during the catalytic cycle of DNA ligases. During DNA binding the OBD translocates by >60 Å and rotates nearly 180° around a swivel point, in order to fit into the minor groove of the DNA substrate. No archaeal DNA ligases have had their structures solved in complex with DNA; however, OBDs have been captured adopting three different conformations (Figure 1). The* S. solfataricus* enzyme exhibited an open and extended conformation in which the OBD was turned away from the AdD (Figure 1(a)); the overall structure resembled that of the DNA ligase from bacteriophage T7 [36]. In contrast, the* Thermococcus* sp. 1519 ligase structure (Figure 1(b)) adopted an intermediate conformation in which the OBD was rotated anticlockwise around the AdD by ~90° compared to the open extended conformation, although this rotation was insufficient to introduce any hydrogen bonds or salt bridges between the OBD and the other domains. A further 120° rotation of the OBD yields a closed conformation, as observed in the structures of the DNA ligases from* P. furiosus* (Figure 1(c)),* A. fulgidus,* and* T. sibiricus*. In these structures a C-terminal helix (Figure 1), found after conserved motif VI, stabilises the closed conformation by mediating several ionic interactions between the OBD and the AdD [26]. This additional helix occupies the cleft between the AdD and OBD in the archaeal unbound structures, but it is displaced in the DNA-bound structure of human DNA ligase I [33].

The domain arrangements of the archaeal ligase structures all differ substantially from those of the DNA-bound structures obtained for human DNA ligases I and III [33, 51], where the three domains encircle the DNA substrate (Figure 1(d)). The emerging picture is one in which conformational flexibility is critical for the correct functioning of archaeal DNA ligases. However, to date, no structures of archaeal DNA ligases in complex with DNA have been solved; therefore it is unknown whether the differences in unbound structures (Figures 1(a)–1(c)) correlate with differences in domain orientations when the DNA substrate is bound. The protein dynamics associated with catalysis at the growth temperatures of the host cells (70–100°C) also remain to be elucidated.

## 4. Biotechnological Applications of Archaeal DNA Ligases

Given their primary physiological role in DNA repair, it is unsurprising that most archaeal DNA ligases have only been assayed for their ability to seal single-stranded nicks in double-stranded DNA. Of more interest for biotechnological applications is the ability to ligate double-stranded, cohesive-, or blunt-ended fragments. These activities have been reported for four archaeal DNA ligases. The enzymes from* Aeropyrum pernix*,* Staphylothermus marinus*,* Thermococcus* sp. 1519, and* T. fumicolans* have all been shown to perform ligation of cohesive-ended fragments. In addition, the* S. marinus* and* T. fumicolans* DNA ligases could also join blunt-ended fragments [19, 40]. Thus, it seems likely that further characterisation of archaeal DNA ligases should yield a pool of enzymes with potential utility in molecular biology and biotechnology.

The most immediate applications for archaeal DNA ligases are likely to be those that exploit their high temperature optima (typically 50–100°C; Table 1). For example, the DNA ligase from* Thermococcus* sp. 1519 is most active at 60–70°C and it is able to ligate DNA fragments with long cohesive ends (12-nucleotide overhangs), but not fragments with shorter (4-nucleotide) cohesive ends or with blunt ends [48]. While it remains to be tested, this combination of properties would appear to make it a promising tool for Gibson assembly [12]. This protocol has rapidly emerged as the dominant method for restriction enzyme-independent assembly of DNA fragments in synthetic biology and it is currently performed at 50°C. We speculate that archaeal DNA ligases, such as the one from* Thermococcus* sp. 1519, may drive the development of new methods, with the promise that ligation at higher temperatures (60–70°C) would decrease the number of incorrect ligation events that arise from misannealing of fragments with short overhangs.

In a similar vein, the DNA ligase from* Staphylothermus marinus* has a half-life of almost 3 h at 100°C and catalyses a variety of ligation reactions with cohesive- and blunt-ended fragments [40]. This extremely thermostable enzyme could find utility in the ligase chain reaction (LCR) for detection of single nucleotide polymorphisms, as it is able to survive the high temperature denaturation steps (~95°C) in the thermal cycling protocol. More generally, it has been shown that thermostable proteins are ideal starting points for protein engineering, as they are more tolerant of mutations and thus yield more functional variants on mutation than their mesophilic homologues [52].

## 5. Engineering an Improved Archaeal DNA Ligase

Despite the ubiquity of DNA ligases in molecular biology, very few attempts have been made to enhance their properties by protein engineering. To date only the DNA ligase from bacteriophage T4 [53] and one archaeal DNA ligase, from* Pyrococcus furiosus* [27, 47], have been targeted.

Nishida and colleagues have successfully used their structural insights [26] to enhance the activity of the* P. furiosus* DNA ligase through structure-guided mutagenesis. In particular, they have targeted the C-terminal helix that interacts with the OBD and the AdD to stabilise the closed conformation of the enzyme (Figure 1(c)). To begin, five polar residues from the OBD (Asp540, Arg544, Gln547, Lys554, and Lys558), each of which contributed to interactions with the AdD, were mutated to alanine [47]. The hypothesis was that destabilising the interdomain interaction would facilitate increased motion of the OBD and thus increase activity by “unlocking” the enzyme. Of the five selected residues, mutation of Asp540, located at the N-terminus of the helical extension, exerted the greatest effect. Further mutagenesis at this position showed that the Asp540→Arg (D540R) substitution gave optimal activity, over a broadened temperature range (20–80°C).

In proof-of-concept ligation-amplification experiments, the authors showed that the engineered ligase (with the D540R mutation) outperformed the wild type at two temperatures. At 60°C, maximum amplification of ligated DNA product was achieved after only 3 cycles with the mutant but took 10 cycles with the wild type enzyme. At 30°C, the engineered enzyme gave maximum product yield after 5 cycles of ligation-amplification, whereas the product yield with the wild type ligase was only ~30% as great, even after 10 cycles [47]. Further, a series of insightful biophysics experiments showed that the introduction of a positively charged arginine residue, in place of the negatively charged Asp540, had accelerated both the formation of the covalent ligase-AMP intermediate and binding of the nicked DNA substrate [47]. This work is also the basis of a granted US patent, number 8,137,943.

In a follow-up study, the same group has recently used further mutagenesis to eliminate the ionic interactions between the AdD and the OBD entirely [27].* P. furiosus* DNA ligase carrying either three point mutations (D540R/K554A/K558A), or D540R plus deletion of the final four amino acids of the C-terminal helix, had further enhanced nick-joining activities. While it is yet to be tested with other archaeal DNA ligases, this design approach, releasing the interactions of the C-terminal helix with the AdD and OBD domains, appears as though it could be a generally applicable one for enhancing activity.

## 6. RNA Ligases

RNA ligases (EC 6.5.1.3) are RNA end-joining enzymes involved in RNA repair, splicing, and editing pathways. In contrast to the ubiquitous DNA ligases, RNA ligases have a narrower phylogenetic distribution. Sequence similarity searches have found RNA ligases in all three domains of life but only in a subset of species [54].

RNA ligases are typically classified into two broad families. The Rnl1 family includes the eponymous RNA ligase 1 (Rnl1) from bacteriophage T4 [3] and the tRNA ligases from fungi, yeasts, and plants [5, 55, 56]. These enzymes repair breaks that have been introduced into single-stranded RNA by site-specific nucleases. The Rnl2 family contains the bacteriophage T4 RNA ligase 2 (Rnl2) and the RNA-editing ligases from the protozoans* Trypanosoma* and* Leishmania*. These enzymes are primarily associated with sealing nicks in RNA made duplex by the presence of a bridging complementary strand [54, 57, 58]. While the RNA ligases share the same six conserved nucleotidyl transferase motifs as DNA ligases, their overall levels of sequence conservation are low. In general, this makes family classification both more difficult and less meaningful.

Like DNA ligases, RNA ligases are also important in molecular biology. T4 RNA ligases 1 and 2 have become essential for a subset of rapid amplification of cDNA ends (RACE) protocols, 3′ RNA labelling, and most importantly, at present, the preparation of microRNA (miRNA) sequencing libraries. ATP-dependent RNA ligases capable of forming phosphodiester bonds between 5′-phosphate and 3′-hydroxyl termini are of most use in these protocols; therefore, they will be the focus of the following sections. For completeness, we also note that two noncanonical RNA ligases from the archaeal species* Pyrococcus horikoshii* have also been reported. The first is a putative 2′-5′ RNA ligase, the structure of which has been solved [59]. The second, RtcB, is an unusual ligase that joins either 2′,3′-cyclic phosphate or 3′-phosphate termini to 5′-hydroxyl termini. Its structure, its mechanism, and its interaction with a novel protein cofactor (Archease) have recently been characterised in detail [60, 61].

## 7. Archaeal RNA Ligases

The first detailed biochemical characterisation of an archaeal RNA ligase was reported in 2008, when an open reading frame from* Pyrococcus abyssi*, previously annotated as encoding a DNA ligase, was found to encode an RNA ligase instead [62]. Previously, it had been assumed that archaeal RNA ligases were likely to be Rnl2-like enzymes, as they showed similarly variant nucleotidyl transferase motifs as T4 Rnl2 [54]. However, the structure of the* P. abyssi* RNA ligase was a marginally closer structural homologue of T4 Rnl1 (secondary structure matching *Z*-score of 6.4, and RMSD of 2.78 Å over 200 aligned residues) than T4 Rnl2 (*Z*-score 6.2, RMSD 2.39 Å over 164 aligned residues) [62]. Further, the* P. abyssi* RNA ligase was active with single-stranded RNA substrates, but not double-stranded RNA, similar to T4 Rnl1.

**Figure 2:**
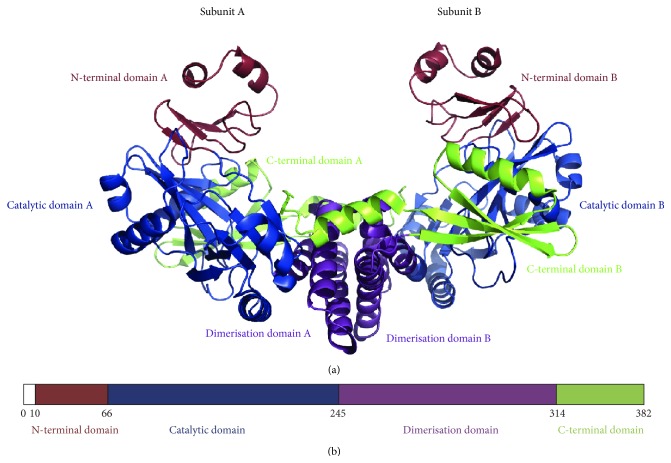
The* Pyrococcus abyssi* RNA ligase. (a) The homodimeric structure of the enzyme (PDB ID 2VUG). The four domains of each monomer are coloured individually and labelled. (b) Schematic diagram of the domain boundaries, with amino acid numbering shown.

Unlike the monomeric mesophilic ligases, X-ray crystallography revealed a homodimeric structure for the* P. abyssi* RNA ligase [62]. Each monomer comprised four domains: an N-terminal domain, a catalytic domain, a dimerisation domain, and a C-terminal domain (Figure 2). The catalytic domain showed structural similarities with other members of the nucleotidyl transferase superfamily. The N-terminal domain resembled that of T4 Rnl1 and has only been observed in these two enzymes to date. The C-terminal domain was all *α*-helical, had no structural homologues, and was not ascribed a function. The dimerisation domain had structural similarity to the copper-binding domain of the amyloid precursor protein, although the metal binding residues are absent in the ligase [62].

The role of dimerisation in the* P. abyssi* RNA ligase is not known. It has been proposed that it may be functionally important for facilitating two symmetric and simultaneous ligation events, such as splicing and intron circularisation [62]. More generally, oligomerisation is a common adaptation associated with thermophily in archaeal proteins [64]. This strategy is thought to increase the rigidity of the individual subunits and promote tighter packing of the hydrophobic core.

To date, the only other archaeal RNA ligase to be characterised biochemically is the one from the species* Methanobacterium thermoautotrophicum* [63], which is now generally known as* Methanothermobacter thermautotrophicus* [65]. The properties of the* P. abyssi* and* M. thermautotrophicus* RNA ligases are summarised in Table 2.

## 8. RNA Ligases in Biotechnology

In addition to their roles* in vivo*, RNA ligases have become important tools in molecular biology [66]. Shortly after the discovery of T4 Rnl1, new protocols were established, including 3′-end biotin and fluorophore labelling [67], RNA ligase mediated rapid amplification of cDNA ends (RLM-RACE) [68], oligonucleotide synthesis [69], and 5′ nucleotide modifications of both RNA and DNA [70].

More recently RNA ligases have become essential for constructing sequencing libraries of microRNAs (miRNAs) and other small RNAs. During library preparation, T4 RNA ligases are used to join 5′- and 3′-adaptors to the RNA substrates, so that the adaptor sequences can be used for priming during reverse transcription and PCR [71–73]. The emerging realisation that miRNAs, small regulatory RNAs involved in posttranscriptional regulation [74], have numerous biological functions and whose misregulation have been implicated in a number of diseases [75], has meant that high-throughput screening has become an invaluable tool for both the discovery and profiling of miRNA expression. Therefore RNA ligases capable of producing high quality sequencing libraries, representative of the original miRNA population in a sample, are of great interest.

Unfortunately, it has become increasingly apparent that miRNA sequencing datasets are prone to severe biases [76] and that the adaptor ligation step is a key contributor. One limitation is the unwanted production of circular by-products [66, 76]. The evolutionarily conserved function of RNA ligases* in vivo* is to seal nicks in RNA hairpin loops (such as those in cleaved tRNA molecules).* In vitro*, this results in a propensity to circularise the RNA substrates, preventing adaptor ligation. Another limitation with the T4 RNA ligases is that they are biased towards ligating particular RNA sequences [77, 78] which can lead to the miscalculation of miRNA abundance by up to 4 orders of magnitude [73]. This ligation bias is not a result of primary sequence preference but instead a bias against RNA secondary structure [79]. Therefore there is growing interest in characterising thermostable RNA ligases that are active at temperatures sufficient to denature RNA secondary structures [80].

## 9. Biotechnological Roles for Archaeal RNA Ligases

Archaeal RNA ligases have found some use in molecular biology protocols. The* M. thermautotrophicus* RNA ligase has the ability to adenylate both single-stranded RNA and single-stranded DNA (Table 2) and it has been used to 5′-adenylate single-stranded DNA adapters for use in the construction of miRNA sequencing libraries [81]. Previously either a chemical synthesis protocol [82] or a methodology involving T4 DNA ligase was used for this adenylation step [83]; however, T4 DNA ligase does not accumulate sufficient adenylated products and the synthesis method was expensive. On the other hand, the* M. thermautotrophicus* RNA ligase accumulates high quantities of the adenylated intermediates (AppRNA and AppDNA) when an excess of ATP is used in the reaction, making it an ideal substitute. This enzyme is currently available commercially as a component of a 5′ DNA adenylation kit (from New England Biolabs).

While the* M. thermautotrophicus* RNA ligase is highly active as a 5′ adenylation enzyme, a single point mutation (Lys97→Ala) resulted in an enzyme that was unable to perform adenylation at all, but which retained the ability to form phosphodiester bonds [81]. This has enabled the development of a two-step protocol in which the wild type enzyme is used to adenylate DNA adapters in an initial reaction. The adenylated adaptors can then be incubated with the pool of target miRNA molecules and the mutated ligase. The result is ligation of preadenylated adaptors to the RNA substrates, with no possibility of circularising the (nonadenylated) RNA [81]. The ability of the* M. thermautotrophicus* RNA ligase (and the K97A mutant) to function at 65°C also helps to remove ligation bias associated with RNA secondary structures. In order to implement this protocol, the mutated enzyme is commercially available (as the Thermostable 5′ AppDNA/RNA Ligase from New England Biolabs).

## 10. Concluding Remarks

In this review, we have summarised the current literature on archaeal nucleic acid ligases. We have highlighted the relative dearth of knowledge on these enzymes, while discussing characteristics that are likely to make them valuable additions to the biotechnologist's toolbox in future. In particular, archaeal enzymes are archetypically thermostable. DNA ligases that are stable and active at elevated temperatures are becoming critical for emerging technologies such as Gibson assembly (which underpins synthetic biology) [12], while thermostable RNA ligases offer the promise of constructing unbiased miRNA sequencing libraries [76]. Moreover, the thermostability of archaeal enzymes makes them ideal starting points for protein engineering [52]. Recent experiments to engineer the* Pyrococcus furiosus* DNA ligase [27, 47] and the* Methanothermobacter thermautotrophicus* RNA ligase [81] demonstrate the great potential for further work in this area.

Overall, the pool of archaeal nucleic acid ligases is diverse but currently undersampled. We anticipate that its further exploration will lead to the discovery of new enzymes with favourable properties for molecular biology and biotechnology, which in turn will drive the development of new methodologies.

## Figures and Tables

**Table 1:** Properties of archaeal DNA ligases.

Organism	Growth conditions	UniProt ID	PDB ID	Cofactor	*T*_opt_	Reference
Bacteriophage						
Bacteriophage T4	Mesophile	P00970	—	ATP	37°C	[16, 37]
Crenarchaeota						
*Aeropyrum pernix *	Hyperthermophile	Q9YD18	—	ATP, ADP	70°C	[38]
*Desulfurolobus ambivalens *	Acidophile/thermophile	Q02093	—		NR	[39]
*Staphylothermus marinus *	Hyperthermophile	A3DP49	—	ATP, ADP	75°C	[40]
*Sulfolobus acidocaldarius *	Acidophile/thermophile	Q4JAM1	—	ATP	85°C	[41]
*Sulfolobus shibatae *	Acidophile/thermophile	Q9P9K9	—	ATP, dATP	60–80°C	[42]
*Sulfolobus solfataricus *	Acidophile/thermophile	Q980T8	2HIX, 2HIV	ATP	NR	[28]
*Sulfophobococcus zilligii *	Hyperthermophile	D2CJS7	—	ATP, ADP, GTP	75°C	[22, 29]
Euryarchaeota						
*Archaeoglobus fulgidus *	Hyperthermophile	O29632	3GDE	ATP	NR	[25]
*Ferroplasma acidarmanus *	Acidophile	S0AR65	—	ATP, dATP	30°C	[43]
*Ferroplasma acidiphilum *	Acidophile	Q2PCE4	—	ATP, NAD^+^	40°C	[41]
*Haloferax volcanii* (LigA)	Halophile	D4GYZ4	—	ATP	NR	[10]
*Haloferax volcanii* (LigN)	Halophile	D4GY98	—	NAD^+^	45°C	[10, 44, 45]
*Methanothermobacter thermautotrophicus *	Thermophile	Q50566	—	ATP, dATP	60°C	[46]
*Picrophilus torridus *	Acidophile	Q6L195	—	ATP, NAD^+^	65°C	[41]
*Pyrococcus furiosus *	Hyperthermophile	P56709	2CFM	ATP	55°C	[26, 27, 47]
*Pyrococcus horikoshii *	Hyperthermophile	O59288	—	ATP	70–90°C	[23]
*Thermococcus *sp. 1519	Thermophile	C0LJI8	3RR5	ATP	70°C	[31, 48, 49]
*Thermococcus fumicolans *	Hyperthermophile	Q9HH07	—	ATP, NAD^+^	65°C	[19]
*Thermococcus kodakaraensis *	Thermophile	Q9HHC4	—	ATP, NAD^+^	65°C	[20, 50]
*Thermococcus onnurineus *	Hyperthermophile	B6YTR4	—	ATP, NAD^+^	80°C	[21]
*Thermococcus sibiricus *	Hyperthermophile	C6A2U9	4EQ5	ATP	NR	[30]
*Thermoplasma acidophilum *	Acidophile	Q9HJ26	—	ATP, NAD^+^	65°C	[41]

*T*_opt_: temperature optimum for the DNA ligase *in vitro*; NR: not reported.

**Table 2:** Properties of archaeal RNA ligases.

Organism	UniProt ID	PDB ID	Co-Factor	*T*_opt_	Properties	Reference
*Pyrococcus abyssi *	Q9UYG2	2VUG	ATP	85–90°C	Efficient at forming adenylated RNA but not so efficient at strand joining. Forms circular products.	[62]

*Methanothermobacter thermautotrophicus *	O27289	—	ATP	65°C	Circularises single-stranded RNA and single-stranded DNA.	[63]

*T*_opt_: temperature optimum for the RNA ligase *in vitro*.
